# Effort–Reward Imbalance and Well-Being Among Foreign Domestic Workers in Singapore

**DOI:** 10.1093/geroni/igaf122.2660

**Published:** 2025-12-31

**Authors:** Mayo Ono, Yasuhiko Saito, Ayumi Honda, Sumihisa Honda

**Affiliations:** Nagasaki University, Nagasaki, Nagasaki, Japan; Nihon University, Tokyo, Tokyo, Japan; St. Mary’s College, Kurume, Fukuoka, Japan; Nagasaki University, Nagasaki, Nagasaki, Japan

## Abstract

Foreign domestic workers (FDWs) play a crucial role in providing long-term care to older adults in Singapore. This study aimed to examine the associations between (1) Effort–Reward Imbalance (ERI) and well-being among FDWs, and (2) ERI and demographic characteristics. This cross-sectional study utilized a structured, self-administered online survey. The participants were 297 female FDWs from the Philippines and Indonesia (age range, 23–59 years) who were providing live-in care for older adults in Singapore. The short version of the Effort–Reward Imbalance Questionnaire and the World Health Organization Five Well-Being Index (WHO-5) were conducted. The results showed that 55.2% of the FDWs had an effort–reward ratio > 1, indicating that their effort exceeded their reward, and 48.5% had a WHO-5 score < 13, suggesting lower well-being. Bivariate analysis revealed that a higher effort–reward ratio was associated with younger age (P = 0.005), higher education (P = 0.018), being unmarried (P < 0.001), less work experience (P = 0.03), providing care for Chinese Singaporeans (P = 0.009), giving dementia care (P = 0.037), a lack of training (P = 0.008), a lack of support (P < 0.001), and lower well-being (P < 0.001). These findings suggest that over half of FDWs experience ERI and nearly half experience poor well-being. Therefore, targeted support is needed, including caregiving training, motivational career development, mental health resources, and assistance for those lacking support. In addition, improving working conditions among FDWs is essential.

